# Genome-wide associations and functional gene analyses for endoparasite resistance in an endangered population of native German Black Pied cattle

**DOI:** 10.1186/s12864-019-5659-4

**Published:** 2019-04-08

**Authors:** Katharina May, Carsten Scheper, Kerstin Brügemann, Tong Yin, Christina Strube, Paula Korkuć, Gudrun A. Brockmann, Sven König

**Affiliations:** 1 Institute of Animal Breeding and Genetics, Justus-Liebig-University of Gießen, 35390 Gießen, Germany; 2 Institute for Parasitology, Center for Infection Medicine, University of Veterinary Medicine Hanover, 30559 Hannover, Germany; 3 Department for Crop and Animal Sciences, Breeding Biology and Molecular Genetics, Faculty of Live Science, Humboldt-Universität of Berlin, 10115 Berlin, Germany

**Keywords:** Endoparasite resistance, Genome-wide associations, 2-step-approach, Candidate genes, Physiological pathways

## Abstract

**Background:**

Gastrointestinal nematodes (GIN), liver flukes (*Fasciola hepatica*) and bovine lungworms (*Dictyocaulus viviparus*) are the most important parasitic agents in pastured dairy cattle. Endoparasite infections are associated with reduced milk production and detrimental impacts on female fertility, contributing to economic losses in affected farms. In quantitative-genetic studies, the heritabilities for GIN and *F. hepatica* were moderate, encouraging studies on genomic scales. Genome-wide association studies (GWAS) based on dense single nucleotide polymorphism (SNP) marker panels allow exploration of the underlying genomic architecture of complex disease traits. The current GWAS combined the identification of potential candidate genes with pathway analyses to obtain deeper insights into bovine immune response and the mechanisms of resistance against endoparasite infections.

**Results:**

A 2-step approach was applied to infer genome-wide associations in an endangered dual-purpose cattle subpopulation [Deutsches Schwarzbuntes Niederungsrind (DSN)] with a limited number of phenotypic records. First, endoparasite traits from a population of 1166 Black and White dairy cows [including Holstein Friesian (HF) and DSN] naturally infected with GIN, *F. hepatica* and *D. viviparus* were precorrected for fixed effects using linear mixed models. Afterwards, the precorrected phenotypes were the dependent traits (rFEC-GIN, rFEC-FH, and rFLC-DV) in GWAS based on 423,654 SNPs from 148 DSN cows. We identified 44 SNPs above the genome-wide significance threshold (*p*_Bonf_ = 4.47 × 10^− 7^), and 145 associations surpassed the chromosome-wide significance threshold (range: 7.47 × 10^− 6^ on BTA 1 to 2.18 × 10^− 5^ on BTA 28). The associated SNPs identified were annotated to 23 candidate genes. The DAVID analysis inferred four pathways as being related to immune response mechanisms or involved in host-parasite interactions. SNP effect correlations considering specific chromosome segments indicate that breeding for resistance to GIN or *F. hepatica* as measured by fecal egg counts is genetically associated with a higher risk for udder infections.

**Conclusions:**

We detected a large number of loci with small to moderate effects for endoparasite resistance. The potential candidate genes regulating resistance identified were pathogen-specific. Genetic antagonistic associations between disease resistance and productivity were specific for specific chromosome segments. The 2-step approach was a valid methodological approach to infer genetic mechanisms in an endangered breed with a limited number of phenotypic records.

**Electronic supplementary material:**

The online version of this article (10.1186/s12864-019-5659-4) contains supplementary material, which is available to authorized users.

## Background

Endoparasite infections imply impaired cattle health and increasing economic losses in pasture-based production systems [1, 2]. Gastrointestinal nematodes (GIN), the bovine lungworm (*Dictyocaulus viviparus*) and the liver fluke (*Fasciola hepatica*) are the most important parasitic helminths of pastured dairy cows [3, 4]. Subclinical infections were associated with reduced milk production [5, 6], impaired reproductive performance [7] and a decrease in product quality [8].

From a farm management perspective, prophylactic as well as diagnosis-based anthelmintic treatments can be applied to control endoparasite infections in affected dairy herds [4, 9]. However, anthelmintic treatments are expensive, and drug residues might pollute the environment and food products [10]. Furthermore, anthelmintic treatments contribute to anthelmintic resistance [11–13]. Hence, sustainable endoparasite control implies the consideration of proper breeding and selection strategies [9, 14]. In this regard, breeding approaches have focused on the selection of specific breeds or genetic lines representing enhanced resistance against specific endoparasites [15, 16].

In cattle, heritability estimates ranged from 0.04 to 0.36 for various definitions of GIN and *D. viviparus* infections [e.g., fecal egg count (FEC), antibody level], indicating a genetic component for pathogen-specific susceptibility [17, 18]. For the liver fluke *F. hepatica*, the heritabilities ranged from 0.09 to 0.33 [15, 19, 20]. The pronounced additive-genetic variances identified were stimuli to explore the underlying genomic architecture for endoparasite resistance in cattle and sheep, with a focus on GIN [21–23]. Genome-wide association studies (GWAS) using dense single-nucleotide polymorphism (SNP) marker panels and QTL mapping approaches contributed to the identification of candidate genes related to immune mechanisms (e.g., the *IFNγ* gene, major histocompatibility complex (MHC)-related genes) against GIN infections in cattle and sheep [24–27]. Coppieters et al. [27] based their studies on microsatellite mapping in Dutch HF cows, and they identified two genome-wide significant QTL on BTA 9 and on BTA 19 influencing FEC for GIN infections. In an experimental Angus population and using microsatellite markers, genome-wide suggestive QTL on BTA 8 and potential linkage with segments on BTA 4, 12 and 17 were associated with patent GIN infections [28]. A GWAS based on 50,000 SNPs identified 12 genomic regions on BTA 3, 5, 8, 15 and 27 as contributing to FEC variation in Angus cattle [29]. Potential candidate genes were related to immunological pathways, i.e., the toll-like receptor-signaling pathway and the cytokine-cytokine receptor interaction pathway [29]. In Angus cattle, association studies based on copy number variations (CNV) have identified immune-related genes, i.e., the genes involved in GIN resistance mechanisms [23, 30, 31]. Although infections with the liver fluke *F. hepatica* in dairy cattle represent a serious animal health problem worldwide [32], studies with a focus on the identification of genomic variants influencing *F. hepatica* resistance are lacking. In sheep, a QTL microsatellite mapping study [33] detected QTL for resistance against *Fasciola gigantica* on OAR 10, 13, 17, 18 and 19, 22.

In Europe, the rising importance of maintaining dairy cattle in grassland systems implies exposure to endoparasite infections and further pathogenic agents [34]. Thus, there is increasing interest in local breeds being best adapted to harsh environments and being less susceptible against infections. The local dual-purpose German Black Pied cattle (DSN, German: *Deutsches Schwarzbuntes Niederungsrind*) is the founder breed of the modern Holstein Friesian (HF) cattle, with a long breeding history in the grassland region of East Frisia, Lower Saxony, Germany [35]. DSN are an endangered breed because they are not competitive with HF regarding milk and protein yield. DSN are defined as robust cattle under harsh environmental conditions [36], and they show better female fertility parameters and a better health status for metabolic disorders after calving compared with HF [37]. Susceptibility to endoparasite infections as measured by the levels of endoparasite burden (i.e., resistance) may not reflect the host’s actual ability to limit the impact of endoparasite infections on fitness and production (i.e., tolerance) [38]. Hence, DSN cows with a high FEC for *F. hepatica* and larvae counts for *D. viviparus* had low somatic cell counts [15]. The udder somatic cell count is a commonly used indicator for mastitis and udder health in overall breeding goals [39]. High levels of somatic cells in milk reflect leukocyte recruitment and indicate udder inflammation. Pimentel et al. [40] discussed antagonistic associations among functional traits and between functional traits and productivity on quantitative-genetic and phenotypic scales. However, the correlations were partly favorable when only considering specific important chromosome segments. Hence, a deeper understanding of the physiological or biological trait interactions is imperative in order to infer the antagonistic relationships between resistance against endoparasites and resistance against udder infections.

For small cattle populations, the limited number of records for complex quantitative traits, especially for health traits, is a special challenge in genomic studies [41]. The use of multibreed reference populations to train on data from several breeds simultaneously was suggested to increase genomic prediction accuracies for production traits [42, 43]. However, it remains challenging to harmonize recording schemes for novel traits across country borders. Within countries, a further methodological approach might be a 2-step strategy. Step 1 involves using a larger data pool of phenotypes from several breeds or genetic lines and correcting the data for fixed effects. Afterwards, in step 2, precorrected phenotypes are dependent traits in GWAS, considering just the small population. A similar strategy was applied to infer quantitative-genetic (co) variance components in small datasets including only daughter records from specific sires [44].

The objectives of the present study were i) to identify genome-wide associations for resistance or susceptibility (measured by FEC) to three endoparasite infections (GIN, *D. viviparus* and *F. hepatica*) in the endangered DSN breed using a 2-step approach; ii) to assess annotations to potential candidate genes and to infer physiological pathways; and iii) to estimate how SNP affects the correlations among endoparasite traits and between endoparasite traits with the test-day milk yield and test-day somatic cell count in chromosomal segments with an impact on disease resistance.

## Methods

### Animal ethics statement

The whole genotyping process from tissue sampling up to the SNP database development was embedded in the logistics and infrastructure of the cow genotyping activities in Germany, organized by the German Holstein breeding organization and their participating regional breeding organizations and farmers. This activity is the basis to implement a national cow training set for genomic selection. Farmers agreed to collect small fecal samples for endoparasite determinations. Such fecal collection does not influence the wellbeing of cows. Fecal samples were analyzed in the laboratory of the university. Thus, no ethical approval was required for this study.

### Animals

The study was incorporated in the framework of a ‘pasture genetics project’ established in 2007 in northwestern Germany. In the framework of this project, a sample of 1166 German Black and White dairy cows distributed over 17 grassland farms was used for genetic line comparisons and quantitative-genetic studies [15]. The five Black and White genetic lines included an HF line selected for milk yield (HF milk); an HF line suited for grazing conditions (HF pasture); a New Zealand HF line (HF NZ); crosses between HF with Jersey, Angler or beef cattle sires (HF cross); and DSN. All cows were exposed to endoparasite infections (access to pasture before 1st of June with > 8 h per day) and not treated with anthelmintics in the sampling year.

A subset of 148 DSN cows from three different farms was selected for genotyping using a selective genotyping approach. In this regard, the selection criteria were i) the herd prevalence for GIN, as GIN was the endoparasite with the highest prevalence in the initial dataset of 1166 cows, ii) individual parasitological measurements per farm (i.e., considering the extreme phenotypes per herd for GIN), and iii) the pedigree-based genetic relationships. The aim was to minimize the average relationship coefficients between all cows selected for genotyping within and between GIN-infected and GIN-non infected cows.

### Phenotypes

The endoparasite dataset considered FEC for GIN (FEC-GIN) and *F. hepatica* (FEC-FH) as well as fecal larvae counts (FLC) for *D. viviparus* (FLC-DV). Based on the coproscopical results, the predominant morphotype for GIN was strongylid eggs (Trichostrongylidae or *Oesophagostomum* and *Bunostomum* spp., respectively) followed by *Strongyloides papillosus* and *Capillaria* spp. eggs (see May et al. [45]). The whole dataset (*n* = 1166 cows) considered repeated measurements for 840 cows. The endoparasite trait definitions in the laboratory are described by May et al. [15]. The test-day production traits included repeated measurements from the whole lactation of the sampling year. Cows with less than five test-day records were excluded from the analysis. The somatic cell counts were log-transformed into somatic cell score: SCS = log_2_ (SCC/100.000) + 3 [46]. Descriptive statistics for the endoparasite traits (FEC-GIN, FEC-FH, FLC-DV) and test-day traits (MY, SCS) for all Black and White cows (whole dataset, n = 1166 cows) and for the genotyped DSN cows (genotype dataset, *n* = 148) are displayed in Table 1.

**Table 1:** Descriptive statistics for endoparasite traits for all cows and for genotyped DSN cows

Endoparasite trait^a^	No. of observations	No. of cows	Mean	SD	Min.	Max.
All cows (*n* = 1166)						
FEC-GIN	1997	1166	11.35	22.57	0	225.0
FEC-FH	2006	1166	0.61	3.64	0	89.0
FLC-DV	1988	1163	0.17	2.14	0	46.0
MY^b^	10,132	1049	22.06	6.98	2.20	57.20
SCS^c^	10,115	1049	3.02	1.64	0.01	10.01
Genotyped cows (*n* = 148)						
FEC-GIN	256	148	15.53	29.80	0	225.0
FEC-FH	256	148	0.57	2.06	0	16.0
FLC-DV	255	148	0.56	4.20	0	45.0
MY	1376	148	19.98	5.77	4.30	47.90
SCS	1369	148	3.07	1.51	0.01	8.60

^a^FEC-GIN fecal egg count for gastrointestinal nematodes, FEC-FH fecal egg count for *Fasciola hepatica*, FLC-DV fecal larvae count for *Dictyocaulus viviparus*

^b^MY Milk yield (kg/cow/day)

^c^SCS somatic cell score (log-transformed somatic cell count: log_2_ (SCC/100.000) + 3)

### Genotypes and quality control

The DSN cows were genotyped using the BovineSNP50 Bead Chip V2 (50k SNP chip) following the Illumina Infinium assay protocol (Illumina Inc., San Diego, CA, USA). In the next step, the genotypes were imputed into Illumina HD Bead Chip level (700 k SNP chip) using a multibreed reference panel of 2188 animals. The reference panel considered 48 DSN animals genotyped with the Illumina HD 700 k Bead Chip array (Illumina Inc., San Diego, CA, USA) and 2140 sequenced animals (including 30 sequenced DSN animals) from the 1000 bull genome project database [47] downscaled to Illumina HD Bead Chip density. Imputation was performed using BEAGLE 4.1 software [48]. The average imputation accuracy from a leave-one-out approach [49] was 89.3%. Only SNP markers on autosomes with validated locations (i.e., based on BLAST analysis against the bovine genome assembly UMD3.1) were considered [49]. The imputed dataset included 587,615 SNP markers from 148 DSN cows. Quality control of the imputed genotype data was performed using the software package PLINK, version 1.9 [50]. SNP markers with a minor-allele frequency (MAF) < 0.05, significant deviation from Hardy-Weinberg equilibrium (HWE, *p* < 10^− 6^) or a call rate < 95% were discarded. Individuals with a call rate < 95% were also excluded. After quality control, the final dataset for GWAS contained 423,654 SNP marker genotypes.

Potential stratification in the dataset due to relatedness among sampled individuals was examined prior to the GWAS using a principal component analysis (PCA). The PCA based on the variance-standardized relationship matrix was derived from the SNP markers as implemented in the --pca option in PLINK.

### Statistical models

#### Precorrection of phenotypic data

In the first step, phenotypes for the endoparasite traits (FEC-GIN, FLC-DV, FEC-FH) and the test-day traits MY and SCS were precorrected for fixed effects using the initial dataset from all 1166 Black and White cows (consideration of all genetic lines). Precorrection of the endoparasite traits for fixed effects was accomplished via a linear mixed model analysis (model 1) using the statistical software SAS, version 9.4 [51] (SAS PROC MIXED, ML method):

1 $$ {y}_{ijklm}=\mu +{F}_i+{P}_j+{GL}_k+{SP}_l+{LS}_m+{e}_{ijklm} $$

*y*_*ijklm*_ = observations for FEC-GIN, FEC-FH and FLC-DV; *μ* = overall mean effect; *F*_*i*_ = fixed effect of the *i*^th^ farm (*i* = 1, …, 17)*; P*_*j*_ = fixed effect of the *j*^th^ parity number (*j* = 1, 2, 3, 4, > 4)*; GL*_*k*_ = fixed effect of the *k*^th^ genetic line (*k* = HF milk, HF pasture, HF NZ, DSN, HF cross)*; SP*_*l*_ = fixed effect of the *l*^th^ sampling period (*l* = June/July, September/October)*; LS*_*m*_ = fixed effect of the *m*^th^ lactation stage according to Huth (1995) (*m* = < 14 days in milk (DIM), 14–77 DIM, 78–140 DIM, 141–231 DIM, ≥232 DIM); and *e*_*ijklm*_ = random residual effect.

Hereafter, the precorrected phenotypes (residuals) for the endoparasite traits (FEC-GIN, FEC-FH, FLC-DV) are denoted as rFEC-GIN, rFEC-FH and rFLC-DV, respectively. Precorrected phenotypes were available from 148 genotyped DSN cows. The distribution of residuals for the endoparasite traits was checked and visually inspected. To further validate the precorrection approach, we correlated the estimated breeding values (EBVs) for all three endoparasite traits from the animal models in May et al. [15] with EBVs from animal models based on precorrected phenotypes. For all traits the EBV correlations between models were > 0.95.

Accordingly, the test-day traits were precorrected for fixed effects via linear mixed model applications (model 2) (SAS PROC MIXED, ML method):

2 $$ {y}_{ijklm}=\mu +{HTD}_i+{P}_j+{TS}_k+{YS}_l+{DIM}_m+{e}_{ijklm} $$

*y*_*ijklm*_ = observations for MY and SCS*; HTD*_*i*_ = fixed effect of the *i*^th^ herd-test-date; *P*_*j*_ = fixed effect of *j*^th^ parity number (1, 2, 3, 4, > 4)*; TS*_*k*_ = fixed effect of *k*^th^ time span between each test-day record and the endoparasite sampling date (≤ − 200, > − 200 and ≤ − 100, > − 100 and ≤ 0, > 0 and ≤ 100, > 100); *YS*_*l*_ = fixed effect of *l*^th^ year-season of last calving (spring, summer, autumn, winter within each year); *DIM*_*m*_ = covariate for days in milk modeled with Legendre polynomials of order 3; and *e*_*ijklm*_ = random residual effect. Hereafter, the precorrected phenotypes (residuals) for the test-day MY and SCS are denoted as rMY and rSCS, respectively.

### Genome-wide association analyses

In the second step, precorrected phenotypes (i.e., residuals from step 1: rFEC-GIN, rFEC-FH, rFLC-DV, rMY, rSCS) were used as dependent variables in single-trait GWAS as implemented in the software package GCTA [52]. All association analyses were performed using the --mlma option in GCTA. The following statistical model for testing single-locus SNP effects was applied:

$$ \boldsymbol{y}=\mathbf{1}\boldsymbol{\mu } +\boldsymbol{xb}+\boldsymbol{u}+\boldsymbol{e} $$

where ***y*** = vector of precorrected phenotypes (rFEC-GIN, rFLC-DV, rFEC-FH, rMY and rSCS); ***μ*** = the overall mean; ***b*** = additive fixed effect of the candidate variant tested for association; **x** = vector of genotypes for the candidate SNP; $\boldsymbol{u}\sim N\left(0,\boldsymbol{G}{\sigma}_u^2\right)$ = vector of random polygenic effects; **G** = genomic relationship matrix (GRM); ${\sigma}_u^2$ = polygenic variance estimated from a null model (i.e., *y* = 1*μ* + *u* + *e*); and $\boldsymbol{e}\sim N\left(0,\boldsymbol{I}{\sigma}_e^2\right)$ = vector of random residual effects, where **I** = an identity matrix and ${\sigma}_e^2$ = the residual variance.

An adjusted Bonferroni correction was applied to account for multiple testing. The traditional Bonferroni correction (i.e., relating the genome-wide significance threshold of 0.05 to the total number of SNP) tends to produce many false-negative results [53]. Therefore, the effective number of independent SNP markers in the analysis (*n* = 111,901) was estimated based on the LD between markers using the software GEC [54]. The adjusted Bonferroni-corrected genome-wide significance threshold with (*p* = 0.05 / n) was *p*_Bonf_ = 4.47 × 10^− 7^. In addition, we considered a chromosome-wide significance threshold (*p*_Cand_ = 0.05 / n_c_), where n_c_ is the effective number of independent SNP markers of the respective chromosome. In this regard, we applied GEC [54]. Chromosome-wide significance thresholds ranged from 7.47 × 10^− 6^ (BTA 1) to 2.18 × 10^− 5^ (BTA 28) (Additional file 1: Table S1).

### Candidate gene annotation and pathway analyses

The biomaRt package [55, 56] from the Bioconductor project was applied to retrieve ‘rs accession numbers’ of associated SNP markers using the getBM() function. Potential candidate genes were queried and assigned to associated SNP markers using the current gene annotations from the ENSEMBL (Version 90) [57] and NCBI (Version 105) [58] databases. A gene was considered as a candidate gene if at least one associated SNP marker above *p*_Cand_ was positioned i) in the respective gene and/or ii) within 5 kb up- and downstream of the respective candidate gene. Regions including the candidate gene ±5 kb up- and downstream are hereafter referred to as regions of interest (ROI). The potential candidate genes identified were manually submitted to the DAVID database (Version 6.8) [59] for pathway and enrichment analyses. In addition, physiological functions and positions of potential candidate genes were further manually reviewed in the KEGG [60], ENSEMBL [57] and NCBI [58] databases.

### Calculation of SNP effect correlations between traits

SNP effect correlations were calculated i) among rFEC-GIN, rFEC-FH and rFLC-DV for the respective potential candidate genes for each trait within all identified ROI and ii) within identified ROI for rFEC-GIN, rFEC-FH and rFLC-DV with rMY and rSCS. SNP effects were not correlated for four ROI for rFLC-DV (corresponding genes: *FAM124B*, *ISL2*, *RCN2*, and *SCAPER*) due to the limited number of marker associations (see Table 2) or identical SNP effects within traits (no variance for the respective ROI).

**Table 2:** Potential candidate genes related to the identified regions associated with endoparasite resistance traits

BTA	Position^a^	No. of associations (total no. of SNP markers)^b^	Position of maximum association (*P*-value)	Gene symbol	Reference^c^
rFEC-GIN					
4	93,956,587 – 94,148,561 ^§^	1 (33)	94,017,547 (1.97 × 10^− 6^) *	*AHCYL2*	ENSBTAG00000001754 | 532836
5	6,777,101 – 7,678,220 ^§^	1 (197)	7,162,997 (3.59 × 10^− 6^) *	*NAV3*	ENSBTAG00000009852 | 528870
18	15,929,200 – 16,156,356 ^§^	1 (10)	16,111,659 (2.79 × 10^− 6^) *	*PHKB*	ENSBTAG00000004806 | 511783
22	890,106 – 1,107,725 ^§^	1 (25)	992,265 (6.41 × 10^− 6^) *	*EGFR*	ENSBTAG00000011628 | 407217
24	61,568,972 – 61,792,092 ^§^	2 (53)	61,565,663 (1.02 × 10^− 5^) *^#^	*PHLPP1*	ENSBTAG00000045832 | 615982
rFEC-FH					
1	50,331,575 – 50,562,844 ^§^	1 (54)	50,365,140 (5.38 × 10^−6^) *	*ALCAM*	ENSBTAG00000000088 | 281614
rFLC-DV					
2	113,274,587 – 113,287,683 ^§^	1 (2)	113,291,934 (2.10 × 10^−9^) **	*FAM124B*	ENSBTAG00000038700 | 506367
	113,369,961 – 113,487,279 ^§^	2 (23)	113,478,686 (6.59 × 10^− 7^) *	*CUL3*	ENSBTAG00000021769 | 534325
5	1,524,095 – 1,565,010 ^§^	3 (13)	1,562,117 (1.07 × 10^−6^) *^#^	*TPH2*	ENSBTAG00000020792 | 100336620
	6,777,101 – 7,678,220 ^§^	2 (197)	6,916,388 (7.69 × 10^–8)^ **	*NAV3*	ENSBTAG00000009852 | 528870
	65,386,934 – 65,454,111 ^§^	1 (4)	65,424,614 (1.29 × 10^−6^) *	*SLC5A8*	ENSBTAG00000011525 | 615734
	68,385,645 – 68,649,661 ^§^	2 (72)	68,420,453 (1.42 × 10^− 6^) *	*CHST11*	ENSBTAG00000010644 | 528860
	97,079,894 – 97,101,600 ^§^	1 (13)	97,105,569 (6.46 × 10^− 6^) *	*EMP1*	ENSBTAG00000036078 | 786490
	97,262,002 – 97,299,627 ^§^	1 (9)	97,265,477 (9.46 × 10^− 7^) *	*FAM234B*	ENSBTAG00000014322 | 512120
	101,572,243 – 101,708,458 ^§^	4 (17)	101,571,894 (8.13 × 10^−6^) *^#^	*RIMKLB*	ENSBTAG00000003291 | 538579
21	12,838,632 – 13,098,198 ^§^	2 (58)	12,918,304 (1.73 × 10^− 7^) **	*MCTP2*	ENSBTAG00000013689 | 532150
	32,107,362 – 32,113,036	1 (1)	32,113,699 (8.56 × 10^−6^) *	*ISL2*	ENSBTAG00000016651 | 786913
	32,118,844 – 32,548,944 ^§^	24 (26)	32,119,934 (8.56 × 10^− 6^) *^#^	*SCAPER*	ENSBTAG00000007382 | 100140107
	32,577,840 – 32,595,999 ^§^	2 (2)	32,580,916 (8.56 × 10^−6^) *^#^	*RCN2*	ENSBTAG00000015780 | 512717
24	7,684,880 – 8,100,543 ^§^	4 (113)	8,077,784 (2.71 × 10^−9^) **	*DOK6*	ENSBTAG00000046957 | 100336967
	21,641,932 – 21,691,820 ^§^	2 (8)	21,676,840 (5,41 × 10^−6^) *^#^	*GALNT1*	ENSBTAG00000011206 | 104975742
	28,992,666 – 29,241,119	1 (60)	29,087,857 (9.04 × 10^− 6^) *	*CDH2*	ENSBTAG00000021190 | 281062
26	4,520,359 – 5,584,422 ^§^	1 (170)	4,781,510 (6.76 × 10^−6^) *	*PCDH15*	ENSBTAG00000045905 | 100140108

^a^Gene position (start-end) in NCBI annotation build on assembly UMD 3.1.1 (genes with differing start and/or end positions in NCBI 105 and ENSEMBL 90 are denoted by ^§^)

^b^Number of associations that reached the suggestive chromosome-wide significance threshold (*p*_Cand_, range: *p* = 7.47 × 10^− 6^ on BTA 1 to *p* = 2.18 × 10^− 5^ on BTA 28) or the Bonferroni-corrected genome-wide significance threshold (*p*_Bonf_ = 4.47 × 10^− 7^) based on the position of the identified candidate gene ±5 kb up- and downstream

^c^Ensembl ID | Entrez ID

*above *p*_Cand_

**above p_Bonf_

^#^Including several marker associations revealing the same *p*-value, the association bases on the SNP marker with the lowest base pair position

## Results

### Population stratification

Additional file 2: Figure S1 includes the top two PCs plotted against each other to visualize the population structure with additional color representation for i) individual farm affiliations and ii) individual endoparasite phenotypes. The analysis revealed three main clusters within the whole population caused primarily by the three different farms. Relationships were closer between the individuals of farm 1 (41 cows) and farm 2 (66 cows), whereas the individuals of farm 3 (41 cows) were not closely linked among each other or to farm 1 and farm 2 individuals. Generally, we only found slight stratification induced by kinship. Hence, we did not account for population stratification via the consideration of PCs in the models for GWAS.

### Genome-wide association analysis for endoparasite traits

**Fig. 1:**
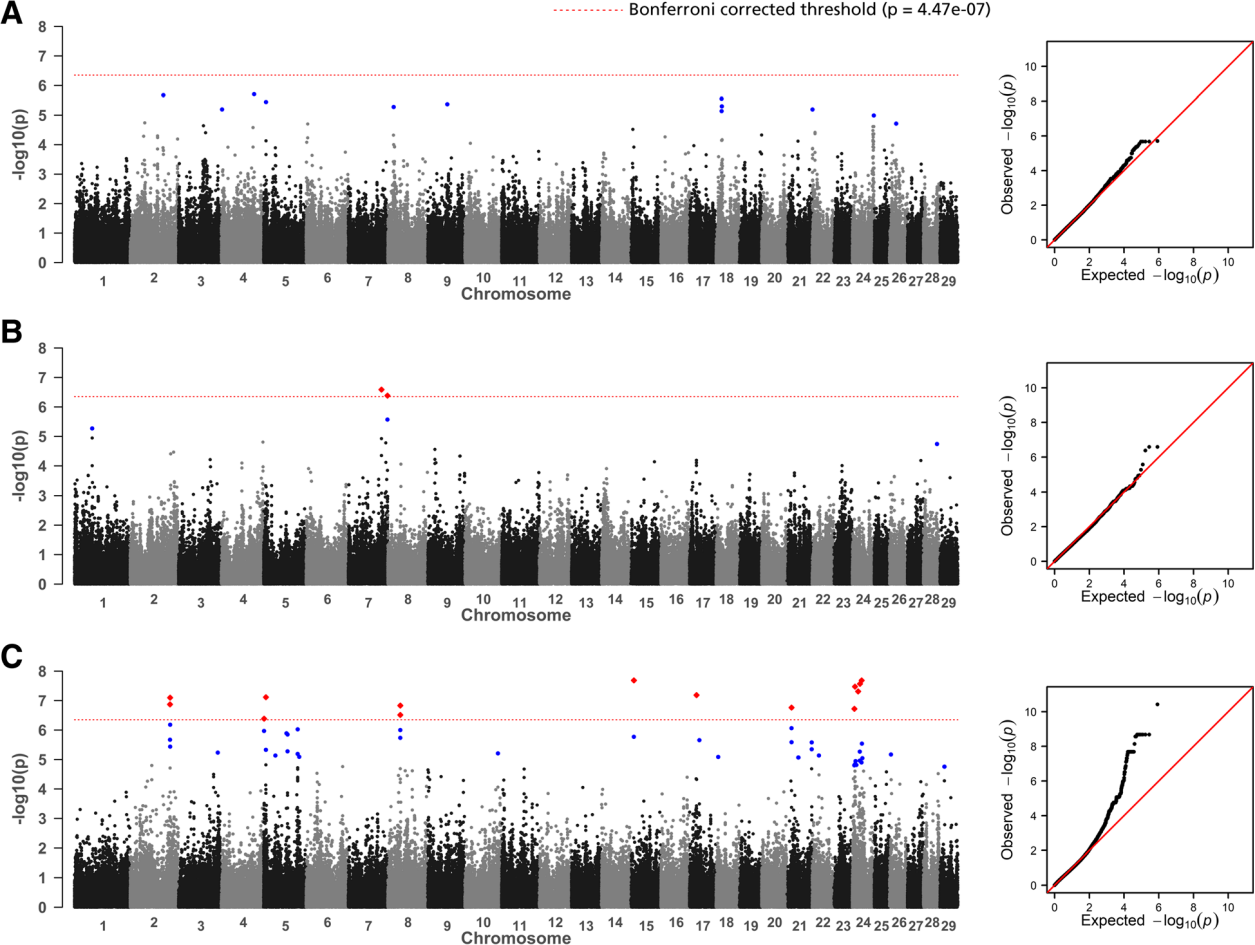
Manhattan plot displaying the GWAS results (*p*-values and corresponding Q-Q plot of observed *p*-values against the expected *p*-values) for **a** rFEC-GIN, **b** rFEC-FH, and **c** rFLC-DV. Bonferroni-corrected genome-wide significance (red line), SNP marker above *p*_Bonf_ (marked in red) and SNP marker above suggestive of the chromosome-wide significance threshold (range: *p* = 7.47 × 10^− 6^ on BTA 1 to *p* = 2.18 × 10^− 5^ on BTA 28) (marked in blue) are also shown

Manhattan plots from the GWAS and corresponding Q-Q plots for rFEC-GIN, rFEC-FH and rFLC-DV are given in Fig. 1. For rFEC-GIN, GWAS identified 17 associated SNP markers based on *p*_Cand_ on 9 chromosomes (Additional file 3: Table S2). None of the SNP markers reached the *p*_Bonf_ level. Most of the associations were detected on BTA 2 (*n* = 4) and BTA 18 (*n* = 3). For rFEC-FH, GWAS identified three SNP markers above *p*_Bonf_ on BTA 7 (Additional file 4: Table S3). In total, three additional variants surpassed the suggestive candidate thresholds *p*_Cand_ on the three chromosomes BTA 1, 7 and 28. GWAS for rFLC-DV identified 41 associations according to the *p*_Bonf_ threshold on BTA 2, 5, 8, 15, 17, 21 and 24 (Additional file 5: Table S4). Moreover, 125 additional variants exceeded the *p*_Cand_ level with a majority (*n* = 44) positioned on BTA 29.

### Gene annotation and pathway analysis

We identified five potential candidate genes for rFEC-GIN (Table 2). More than two neighboring significantly associated SNP markers without any non-associated marker positioned between them were defined as an association cluster. One association cluster including two variants on BTA 24 was related to the *PHLPP1* (PH domain and leucine rich repeat protein phosphatase 1) gene. The *PHLPP1* gene is a protein coding gene involved in immunological processes (PI3K-Akt signaling pathway; KEGG entry: bta04915), e.g., the regulation of T cell energy (Table 3). Functional annotation from rFEC-GIN candidate genes revealed the estrogen signaling pathway (KEGG entry: bta04915) for the candidate gene *EGFR* (Table 3). We identified three immunological pathways for the *EGFR* (Epidermal growth factor receptor) gene, with the regulating cells involved in innate as well as adaptive inflammatory host defenses (Table 3). The *ALCAM* (Activated leukocyte cell adhesion molecule) gene was related to rFEC-FH on BTA 1 (Table 2). This gene was annotated to the (immune) cell adhesion molecules (CAMs) pathway (KEGG entry: bta04514) (Table 3).

**Table 3:** Candidate genes related to pathways potentially associated with endoparasite resistance

Pathway	Endoparasite trait	Candidate gene (BTA)^a^	Possible association to endoparasite infections
Cell adhesion molecules pathway	rFEC-FH	*ALCAM* (BTA1),	Cell adhesion interactions of T cells^b^
	rFLC-DV	*CDH2* (BTA 24)	
Cytokine-cytokine interaction pathway	rFEC-GIN	*EGFR* (BTA 22)	Intercellular regulation and mobilization of adaptive immune response cells^b^
Estrogen signaling pathway	rFEC-GIN	*EGFR* (BTA 22)	Increase in reproduction rate of parasites as a result of increasing metabolism of 17-ß-estradiol in its host [74–76]
PI3K-Akt signaling pathway	rFEC-GIN	*EGFR* (BTA 22), *PHLPP1* (BTA 24)	Important functions in cellular immune response^b^

^*a*^Gene ID (chromosomal location); ^b^Based on annotation in KEGG pathway [60]; BTA Bos taurus chromosome*,* rFEC-GIN Residuals of fecal egg counts for gastrointestinal nematodes*,* rFEC-FH Residuals of fecal egg counts for *Fasciola hepatica*, rFLC-DV Residuals of fecal larvae counts for *Dictyocaulus viviparus*

We detected 17 potential candidate genes for rFLC-DV (Table 2). An association cluster including 24 consecutively positioned and associated SNP markers was detected on BTA 21. This cluster is related to the *SCAPER* (S-Phase cyclin A associated protein in the endoplasmic reticulum) gene. The *GSG1* (Germ cell associated 1) gene and the *RIMKLB* (Ribosomal modification protein RimK-like family member B) gene on BTA 5 revealed association clusters, too. Functional annotation from the rFLC-DV candidate genes revealed the cell adhesion molecules (CAMs) pathway for the *CDH2* gene on BTA 24 (Table 3).

The *NAV3* (Neuron navigator 3) gene was the only candidate gene associated with more than one trait (rFEC-GIN and rFLC-DV). *NAV3* is involved in the regulation of interleukin 2 production by T cells. However, the marker associations within *NAV3* did not overlap between rFEC-GIN and rFLC-DV. For rFEC-GIN, one SNP marker positioned in the middle of *NAV3* was identified above *p*_Cand._ For rFLC-DV, two SNP markers positioned near the gene start position were significantly associated. The space between both association signals for rFEC-GIN and rFLC-DV in *NAV3* was approximately 235 kb.

### SNP effect correlations between endoparasite traits

**Fig. 2:**
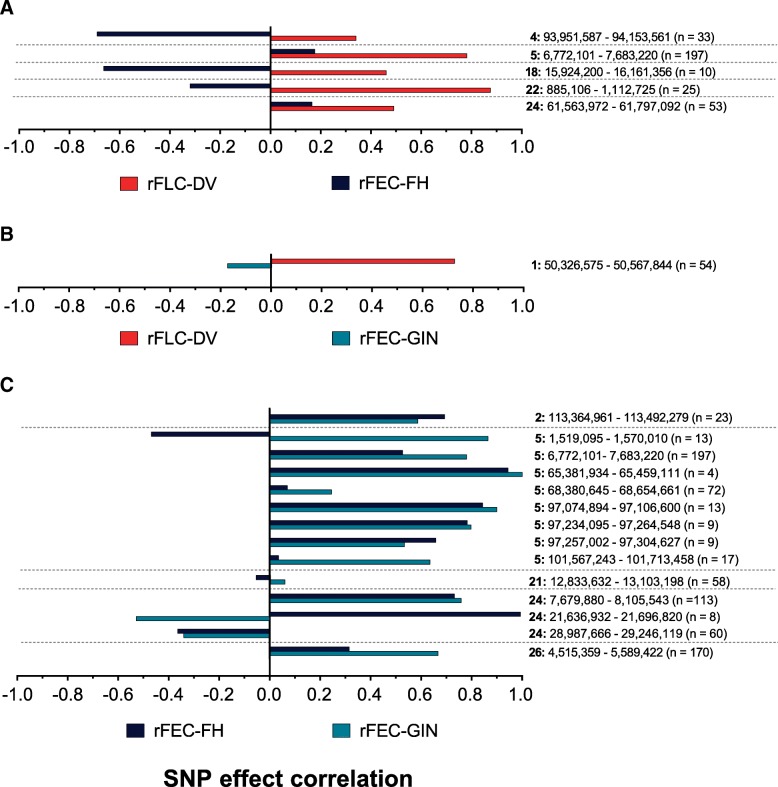
SNP effect correlations between endoparasite traits for the identified genomic regions of potential physiological significance (candidate gene position plus 5 kb up- and downstream) for **a** rFEC-GIN, **b** rFEC-FH and **c** rFLC-DV

**Fig. 3:**
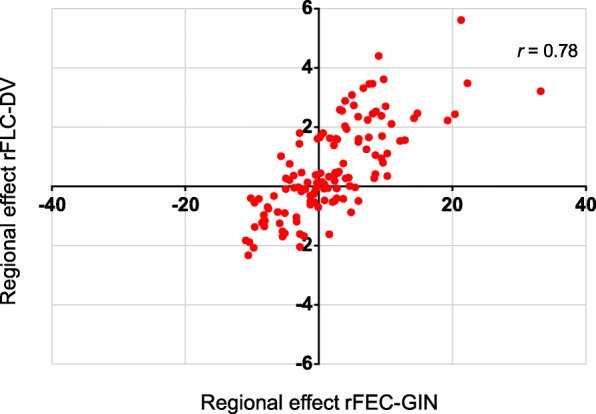
Correlations based on SNP effects between rFLC-DV and rFEC-GIN within the common ROI on BTA 5 (ROI: bp 6,772,101 – 7,683,220; including the *NAV3* gene)

The number of SNP markers within all identified ROI ranged from 8 to 197. For three of the five ROI for rFEC-GIN, we found antagonistic (negative) SNP effect correlations (− 0.32 to − 0.69) between rFEC-GIN with rFEC-FH (Fig. 2). Only on BTA 5 (6,772,101 – 7,683,220) and within the ROI on BTA 24 (bp 61,563,972 – 61,797,092) were correlations slightly positive (Fig. 2). We detected moderate to high SNP effect correlations (0.34 to 0.87) between rFEC-GIN and rFLC-DV within all ROI for rFEC-GIN. The SNP effect correlation between marker effects for rFEC-FH and rFEC-GIN was negative (− 0.17) for the ROI identified for rFEC-FH (Fig. 2). For the same ROI, the correlation between SNP effects for rFEC-FH and rFLC-DV was 0.73 (Fig. 2). Considering the identified ROI for rFLC-DV, correlations ranged from − 0.53 on BTA 24 to 0.99 on BTA 5 between marker effects for rFLC-DV and rFEC-GIN (Fig. 2). The correlations between the marker effects for rFLC-DV and rFEC-FH ranged from − 0.47 on BTA 5 to 0.99 on BTA 24 (Fig. 2). The correlation was 0.78 between the marker effects for rFLC-DV and rFEC-GIN considering the common ROI on BTA 5 (ROI: bp 6,772,101 – 7,683,220; including the *NAV3* gene) (Fig. 3).

### SNP effect correlations between endoparasite traits and test-day traits

**Fig. 4:**
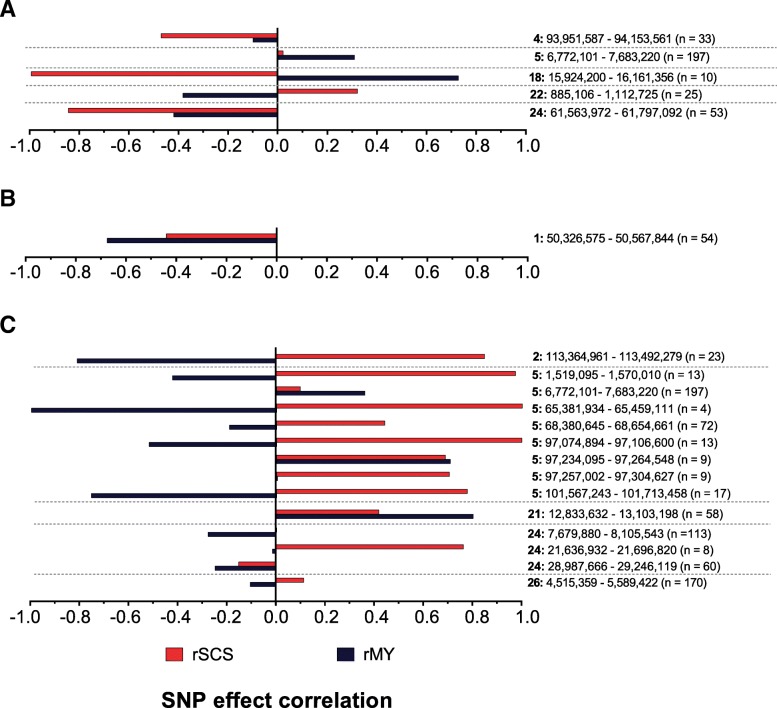
SNP effect correlations between the residuals of endoparasite traits and the residuals of test-day traits somatic cell score (SCS) and milk yield (MY) for the identified genomic regions of potential physiological significance (candidate gene position plus 5 kb up- and downstream) for **a** rFEC-GIN, **b** rFEC-FH and **c** rFLC-DV

The SNP effect correlations between rFEC-GIN, rFEC-FH and rFLC-DV with rMY and rSCS are presented in Fig. 4. The correlations between the marker effects for rFEC-GIN and rMY were negative (− 0.10 to − 0.42) for three ROI. The marker effect correlations between rFEC-GIN and rMY were moderate to large (0.31 to 0.73) for two ROI. Differing correlations between rFEC-GIN and rSCS were estimated for different ROI, i.e., positive correlations (0.02 to 0.32) on BTA 5 and 22 but negative correlations (− 0.47 to − 0.99) on BTA 4, 18 and 24.

Regarding the ROI identified for rFEC-FH, the correlation between the SNP effects for rFEC-FH and rMY was − 0.67, and it was − 0.44 between the SNP effects for rFEC-FH and rSCS. The correlations between the SNP effects for rFLC-DV and rMY were in a positive range (0.00 to 0.80) for four ROI, with the largest correlation on BTA 21. Additionally, differing correlations between rFLC-DV and rMY were detected for different ROI from the same BTA on BTA 5. The SNP effect correlations between rFLC-DV and rSCS were positive (0.10 to 0.99) for 12 ROI and neutral or negative (0.00 to − 0.15) for two ROI. The correlations between rFLC-DV and rSCS differed for different ROI on BTA 24. Positive correlations for different ROI were observed on BTA 5. For seven ROI on BTA 2, 5 and 26, the correlations between the SNP effects for rFLC-DV and rMY ranged from − 0.11 to − 0.99, whereas those with rSCS were positive (ranging from 0.11 to 0.99).

## Discussion

### Genome-wide association analysis for endoparasite traits

In local breeds with a small population size (e.g., DSN), reduced genetic variation and diversity compared with the intensively selected HF breed is reported [61, 62]. Thus, in comparison with HF or beef cattle breeds [27–29], other SNP variants associated with endoparasite resistance have been expected. The cattle selection lines best adapted to harsh grassland environments (e.g., New Zealand HF lines, DSN) [35, 63] are often described as more robust and less susceptible to endoparasite infections [15]. One explanation addresses genetic resistance to disease or endoparasite infections via cellular immunological mechanisms and adaptive immune responses, which differ between breeds or selection lines [9, 64]. Hence, selection signatures were identified when grouping subpopulations according to DSN or HF gene percentages, and when focusing on genomic regions with an impact on disease resistance [65]. In addition, higher levels of genomic homogeneity and of genetic relatedness in DSN than in HF contribute to a decrease in polymorphism, influencing the power to detect marker associations [66].

For rFEC-GIN, the majority of SNP marker associations were detected on BTA 2. Candidate genes were identified on BTA 4, 5, 18, 22 and 24. In contrast, in Angus beef cattle, genomic regions on BTA 3, 5, 15 and 27 were significantly associated (−log_10_ *P* = 4.3) with GIN infections [29]. In Scottish Blackface sheep, evidence for associations with GIN infections were detected on OAR 6 and 14 [66], but the SNPs located on OAR 3 and 12 affected FEC in crossbred sheep (Martinique Black Belly x Romane sheep) [67]. For Dutch Holstein-Friesian dairy cattle, genome-wide suggestive QTL on BTA 11, 14, 21, 24, 25 and 27 were reported using a within-family analysis based on a dataset of 768 phenotyped cows [27]. In the same study, genome-wide significant QTL were identified on BTA 9 and 19 in an across-family analysis [27]. In agreement with the other studies based on FEC, we also detected significantly associated SNPs for GIN resistance (according to *p*_Cand_) on BTA 5, 9 and 24.

The pre-selection of cattle according to phenotypes or allele frequencies, the phenotyping strategy (e.g., utilization of experimental vs. field data) [28, 29], and the differences in trait definitions and parasitological examination techniques are further possible explanations for the different GWAS results in different populations or breeds. In the current study, we did not distinguish among the different species according to the GIN morphotypes. We assumed that the diversity of GIN species in our study follows the distribution usually reported for cattle, with *Ostertagia ostertagi* as the most common species [68] followed by *Cooperia* spp. and *Trichostrongylus* spp. [69]. In field data studies, differences in infection exposure among individuals and environments and the variable infection pressure over time explain the reduced power to detect SNP associations and potentially mask the genetic signals [70–72]. The total number of associations was highest for rFLC-DV although a low prevalence was detected for *D. viviparus* in the multibreed dataset of 1166 cows [15]. False-negative marker associations could be the outcome in a dataset with a low number of infected cows. From this perspective, associations for rFLC-DV should be viewed with caution. However, the low prevalence of *D. viviparus* in our genotyped DSN cows reflected the phenotypic trait distribution in the whole cattle population.

GWAS results for GIN infections in previous studies [27, 29] reflect the findings for rFLC-DV. *D. viviparus* and GIN represent the same biological order (Strongylida). Thus, overlaps in marker associations between both species have been expected. We found signals on seven common chromosomes, with impacts on both the rFLC-DV and rFEC-GIN traits. A large number of associations and potential candidate genes were identified on BTA 2 and BTA 24 for rFEC-GIN and rFLC-DV. The *NAV3* gene on BTA 5 was detected for both traits; however, no same SNP was identified to be simultaneously associated. Different SNP variants for different nematode species [trichostrongylids (herein referred to as GIN) and *Nematodirus* spp., which belong to the same biological order] were detected in Scottish Blackface lambs [66]. Furthermore, a GWAS for ectoparasites (different tick species) in cattle revealed SNP associations on different chromosomes for the ixodid tick species *A. hebraeum* and *R. evertsi evertsi* [73].

Regarding rFEC-FH, the three significant markers based on *p*_Bonf_ were not related to potential candidate genes. Efforts to characterize genes or genomic regions for liver fluke traits in ruminants were reported for *Fasciola gigantica* in sheep [33]*.* Thus, the current findings present a novelty for enhancing disease resistance to *F. hepatica* in cattle breeds.

### Gene annotation and pathway analysis

Our study identified the cytokine-cytokine receptor interaction pathway for the *EGFR* gene for rFEC-GIN. In addition, this pathway was identified in a GWAS for Angus cattle [29]. The most interesting finding was the estrogen signaling pathway involving the potential candidate gene *EGFR* for rFEC-GIN. Experiments in mice have indicated that parasites can exploit the hormonal host microenvironment to favor their establishment, growth and reproduction rate [74, 75]. In this regard, for the tapeworm *Taenia crassiceps,* an increase in the physiological concentrations of the (host) hormone 17-ß-estradiol was associated with an increase in the reproductive capacity of *T. crassiceps* cysticerci [76]. Moreover, steroid hormone synthesis (e.g., progesterone, testosterone) influenced the fertility of *Schistosoma mansoni* and increased the length of *Ascaris suum* larvae in its host [77, 78]. However, it remains unclear whether similar mechanisms of host exploitation via the regulation of host hormones such as estradiol are also due to infections with GIN. The PI3K-Akt signaling pathway was annotated to several candidate genes for rFEC-GIN. There is evidence that the phosphatidylinositol-3 kinase (PI3K) plays a decisive role in cellular immune response, activated by costimulatory receptors of B and T cells in mice [79, 80]. Furthermore, the PI3K-Akt signaling pathway was identified for the protozoa *Neospora caninum*, an intracellular parasite that causes high economic losses in the cattle industry [81].

The activated leukocyte cell adhesion molecule (*ALCAM*) gene on BTA 1 detected for rFEC-FH is related to the cell adhesion molecules (CAMs) pathway, and it plays a crucial role in immune response mechanisms, e.g., cell adhesion interactions of T cells. Interestingly, the same pathway was also detected for *D. viviparus* as being related to the *CDH2* (Cadherin 2, type 1, N-cadherin) gene on BTA 24. Our GWAS revealed several genes and three pathways as being involved in T lymphocyte interactions for rFEC-GIN, rFEC-FH and rFLC-DV. The cellular mechanisms mediated by T lymphocyte recruitment are typical features of immune response to endoparasite (especially helminth) infections in its host [82–84]. In cattle, natural infections with *F. hepatica* induce Th2-associated reactions, with simultaneous inhibition of Th1 cell activity [85]. Immune response against GIN mainly involves Th2 mechanisms in order to decrease the number of adult worms and of FEC. A mixed Th1/Th2 response follows infections with *D. viviparus* in cattle [85]. An interesting finding was made in Nelore cattle, where the immune response to the GIN species *Cooperia punctata* and *Haemonchus placei* was probably mediated by Th2 cytokines in the resistant cattle group and induced by Th1 cytokines in the susceptible ones [86, 87]. Thus, variations in identified immunological pathways might be expected when applying GWAS based on a stringent case-control (resistance and susceptible groups) design. The *CDH2* and *PCDH15* genes on BTA 24 and 26 for rFLC-DV coded for adhesion molecules, and they were expressed in the cattle selected for either resistance or susceptibility to nematode parasites [88].

### Correlations between SNP effects for endoparasite traits

Differing correlations for SNP effects between rFEC-GIN, rFEC-FH and rFLC-DV indicate the complexity of resistance against different infectious agents. In most cases, negative SNP effect correlations were observed between rFEC-FH and rFEC-GIN, implying that genomic selection on improved resistance to *F. hepatica* infections simultaneously increased the susceptibility to GIN. The pedigree-based genetic correlations ranged from − 0.10 to 0.17 between different GIN and liver fluke trait definitions [15, 20]. One explanation for the negative correlations on a genomic scale between rFEC-FH and rFEC-GIN and for the divergent marker associations as well as gene annotations might be due to the variations in immune response mechanisms for different endoparasite species. In cattle, the specific immune response for *F. hepatica* (antibody response of the IgG1, the cellular response associated with the cytokines interleukin IL4, IL10, TNF-ß) differs from those for GIN and *D. viviparus* [89]*,* where the immune response is predominantly mediated by IgA, IgE, IgG and IgM [90, 91].

The correlations based on the SNP effects between the two nematode traits rFEC-GIN and rFLC-DV were positive for most of the identified ROI, confirming the estimates from a quantitative-genetic study [15]. High positive genetic correlations between the different endoparasite species were reported in sheep [92, 93], which simplifies selection strategies.

### Correlations between SNP effects for endoparasite and test-day traits

The SNP effect correlations between rFEC-GIN and rMY were negative for three of the five identified ROI, corresponding to the estimates from pedigree-based random regression models [15]. Twomey et al. [20] detected genetic correlations close to zero between milk yield and antibodies for *Ostertagia ostertagi*, the most common GIN species in cattle. In our study, mostly negative SNP correlations were inferred between rMY and rFLC-DV. Thus, on a genomic scale, breeding for higher milk production reduces larvae shedding of the bovine lungworm. Highly positive SNP effect correlations between rFLC-DV and rMY were detected for ROI on BTA 5 and 21, indicating a coregulation of both traits in these regions. Another possibility is that the genes affecting FLC for *D. viviparus* and MY were in a low linkage disequilibrium.

Regarding the associations between endoparasite traits and SCS, the SNP effect correlations between rFEC-GIN, rFEC-FH and rFLC-DV with rSCS for the ROI partly reflect quantitative-genetic estimates. May et al. [15] estimated positive (i.e., favorable from a breeding perspective) genetic correlations between FEC-GIN and SCS throughout lactation. In contrast, for most of the identified ROI, correlations based on SNP effects were unfavorable between rFEC-GIN and rSCS, but the SNP effect correlations between rFLC-DV and rSCS were positive (i.e., favorable from a breeding perspective). The negative SNP effect correlation between rFEC-FH and rSCS for the ROI on BTA 1 (including the *ALCAM* gene) reflects the pedigree-based estimates, i.e., the negative genetic correlations in the course of lactation [15]. Hence, breeding for reduced FEC for GIN or *F. hepatica* induces an increase in SCS. Vice versa, breeding on low somatic cells implies increasing FEC for GIN and *F. hepatica*. Such findings have practical relevance when developing breeding programs with a focus on both disease resistance and tolerance [94]. In particular, the antagonistic relationship between SCS and egg or larvae counts for endoparasite traits put into question the suitability of SCS as an indicator for udder health. Only moderate phenotypic and genetic correlations between SCS and clinical mastitis, as well as major pathogen susceptibility for cows with extremely low SCS [39], are a further justification in this regard. Mechanisms that decrease somatic cells in milk do not necessarily eliminate the causative pathogens during mastitis [95]. Phenotypically, the correlations between *F. hepatica* infections and SCS were close to zero (− 0.04 to 0.03 for different test-days around the parasitological examination date), reflecting the results from previous studies in HF dairy cow populations [6, 96]. Association analyses between ectoparasite and endoparasite infections with milk composition traits were of great interest in previous studies [20, 97]. Nevertheless, to our knowledge, this is the first approach focusing on the underlying genetic background between endoparasite infections and host defense mechanisms to further pathogen infections (e.g., increase in somatic cells).

## Conclusions

The 2-step approach using precorrected phenotype data based on a larger dataset of related genetic lines was a valid approach to estimating SNP marker effects and to inferring possible candidate genes and biological pathways for endoparasite resistance in a small sample of genotyped dual-purpose DSN cows. Such a methodological approach might be suitable for genomic studies with a focus on novel traits in small populations. In total, 23 potential candidate genes were annotated to SNP marker associations for rFEC-GIN, rFEC-FH and rFLC-DV. A shared ROI (including the *NAV3* gene) was only identified for GIN and *D. viviparus* on BTA 5. Five of the identified possible candidate genes were directly involved in immune response mechanisms. The inferred estrogen signaling pathway is involved in host-parasite interactions, and it appears to be specific for rFEC-GIN. Functional gene annotations identified a common immunological pathway (e.g., cell adhesion molecules pathway for rFEC-FH and rFLC-DV) for different endoparasite traits. The SNP effect correlations between rFEC-GIN and rFLC-DV were quite large for most of the ROI, indicating a partly joint genetic basis for traits representing the same biological order. The negative SNP effect correlation between rSCS and rFEC-FH is in agreement with pedigree-based genetic correlations, and it indicates an antagonistic association between disease resistance for udder and endoparasite infections. Generally, we demonstrated that resistance to the nematodes GIN and *D. viviparus* and to the trematode *F. hepatica* is under polygenic control through a large number of loci with moderate to small effects. The SNP effect correlations for specific endoparasite ROI provided deeper insight into trait associations and contributed to physiological explanations of possible genetic antagonisms between disease resistance and productivity. Predominantly negative SNP effect correlations between GIN or *F. hepatica* with SCS indicate the complexity of immune response mechanisms but also raise critical questions regarding breeding strategies on low somatic cell scores.

## Additional files

Additional file 1: **Table S1.** Chromosome-wide significance thresholds for the endoparasite traits rFEC-GIN, rFEC-FH and rFLC-DV including the number of SNP markers after quality control (QC) and the effective number of independent SNP markers based on linkage disequilibrium (LD). (DOCX 16 kb)

Additional file 2: **Figure S1.** Principal component analysis of the 148 DSN cattle for (A) the mean values of FEC-GIN, (B) the mean values of FEC-FH, (C) the mean values of FLC-DV and (D) the three different farms. Plot of the first two principal components (PC1 and PC2) of each individual cow based on SNP information to evaluate the extent of the population structure. (PDF 31 kb)

Additional file 3: **Table S2.** List of all SNP markers associated with the residuals of gastrointestinal nematodes (rFEC-GIN) identified in Black Pied dairy cattle by genome-wide analysis. (DOCX 19 kb)

Additional file 4: **Table S3.** List of all SNP markers associated with the residuals of *Fasciola hepatica* (rFEC-FH) identified in Black Pied dairy cattle by genome-wide analysis. (DOCX 15 kb)

Additional file 5: **Table S4.** List of all SNP markers associated with the residuals of *Dictyocaulus viviparus* (rFLC-DV) identified in Black Pied dairy cattle by genome-wide analysis. (DOCX 38 kb)

## Abbreviations

BLAST: Basic Local Alignment Search Tool
Bp: Base pairs
BTA: *Bos taurus* chromosome
CAMs: Cell adhesion molecules
CNV: Copy number variations
DSN: Deutsches Schwarzbuntes Niederungsrind (German Black Pied cattle)
EPG: Eggs per gram feces
FEC: Fecal egg count
FEC-FH: Fecal egg count for *Fasciola hepatica* (liver flukes)
FEC-GIN: Fecal egg count for gastrointestinal nematodes
FLC: Fecal larvae count
FLC-DV: Fecal larvae count for *Dictyocaulus viviparus* (bovine lungworm)
GIN: Gastrointestinal nematodes
GRM: Genomic relationship matrix
GWAS: Genome-wide association analysis
HF: Holstein Friesian
HTD: Herd-test date
HWE: Hardy-Weinberg equilibrium
IFNγ: Interferon-Gamma
IgG: Immunoglobulin
KEGG: Kyoto Encyclopedia of Genes and Genomes
LD: Linkage disequilibrium
MAF: Minor allele frequency
Mb: Megabases
MHC: Major Histocompatibility Complex
MY: Milk yield
NCBI: National Center for Biotechnology Information
OAR: *Ovis aries* chromosome
*p*_Bonf_: Bonferroni-corrected genome-wide threshold (*p* = 4.47 × 10^− 7^)
PCA: Principal component analysis
*p*_Cand_: Suggestive chromosome-wide significance threshold (range *p* = 7.47 × 10^− 6^ on BTA 1 to *p* = 2.18 × 10^− 5^ on BTA 28)
QTL: Quantitative trait loci
rFEC-FH: Residuals of FEC-FH
rFEC-GIN: Residuals of FEC-GIN
rFLC-DV: Residuals of FLC-DV
rMY: Residuals of milk yield
rSCS: Residuals of somatic cell score
SCC: Somatic cell count
SCS: Somatic cell score
SNP: Single nucleotide polymorphism
