# Supplementary material for: Genome-wide associations and functional gene analyses for endoparasite resistance in an endangered population of native German Black Pied cattle
Source: BMC Genomics. 2019 Apr 8;20:277. doi: 10.1186/s12864-019-5659-4 (PMC6454736; doi:10.1186/s12864-019-5659-4)
Supplement: Supplementary file 1 — Table S1. Chromosome-wide significance thresholds for the endoparasite traits rFEC-GIN, rFEC-FH and rFLC-DV including the number of SNP markers after quality control (QC) and the effective number of independent SNP markers based on linkage disequilibrium (LD). (DOCX 16 kb) [file 12864_2019_5659_MOESM1_ESM.docx]

Additional file 1. Chromosome-wide significance thresholds for the endoparasite traits rFEC-GIN, rFEC-FH and rFLC-DV including the number of SNP markers after quality control (QC) and the effective number of independent SNP markers based on linkage disequilibrium (LD).

| BTA | SNP marker after QC | Effective number of independent SNP marker based on LD calculated using GEC [54] | *p*-value threshold  *p*_Cand_ |
| --- | --- | --- | --- |
| 1 | 27303 | 6696 | 7.47E-06 |
| 2 | 22710 | 5774 | 8.66E-06 |
| 3 | 20234 | 5103 | 9.80E-06 |
| 4 | 20603 | 5390 | 9.28E-06 |
| 5 | 18810 | 4448 | 1.12E-05 |
| 6 | 22292 | 5816 | 8.60E-06 |
| 7 | 19373 | 4858 | 1.03E-05 |
| 8 | 15643 | 4180 | 1.20E-05 |
| 9 | 17186 | 4158 | 1.20E-05 |
| 10 | 18268 | 4982 | 1.00E-05 |
| 11 | 19296 | 4705 | 1.06E-05 |
| 12 | 14394 | 3927 | 1.27E-05 |
| 13 | 12170 | 3536 | 1.41E-05 |
| 14 | 12850 | 3614 | 1.38E-05 |
| 15 | 13730 | 3620 | 1.38E-05 |
| 16 | 13921 | 3412 | 1.47E-05 |
| 17 | 13356 | 3608 | 1.39E-05 |
| 18 | 11600 | 3142 | 1.59E-05 |
| 19 | 12172 | 3719 | 1.34E-05 |
| 20 | 13822 | 3757 | 1.33E-05 |
| 21 | 11604 | 2887 | 1.73E-05 |
| 22 | 11409 | 3021 | 1.65E-05 |
| 23 | 8689 | 2782 | 1.80E-05 |
| 24 | 10861 | 2815 | 1.77E-05 |
| 25 | 8120 | 2567 | 1.95E-05 |
| 26 | 8933 | 2375 | 2.11E-05 |
| 27 | 8145 | 2346 | 2.13E-05 |
| 28 | 7890 | 2292 | 2.18E-05 |
| 29 | 8270 | 2371 | 2.11E-05 |
| overall | 423654 | 111902 | 4.47E-07 (*p*_Bonf_) |
