# Supplementary figures and images for: Genome-wide associations and functional gene analyses for endoparasite resistance in an endangered population of native German Black Pied cattle
Source: BMC Genomics. 2019 Apr 8;20:277. doi: 10.1186/s12864-019-5659-4 (PMC6454736; doi:10.1186/s12864-019-5659-4)

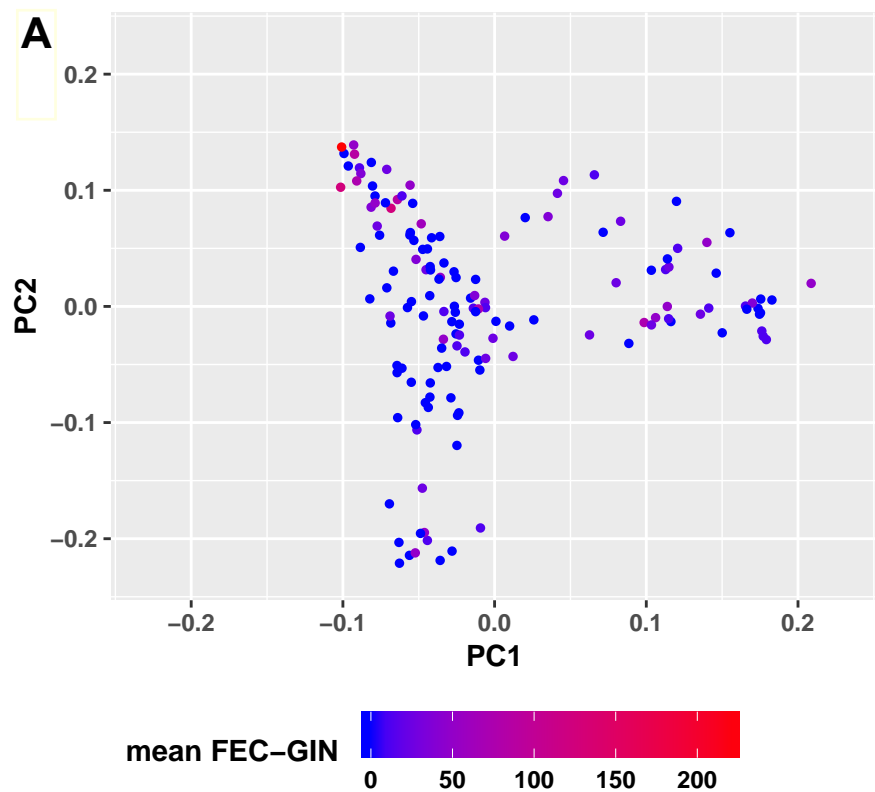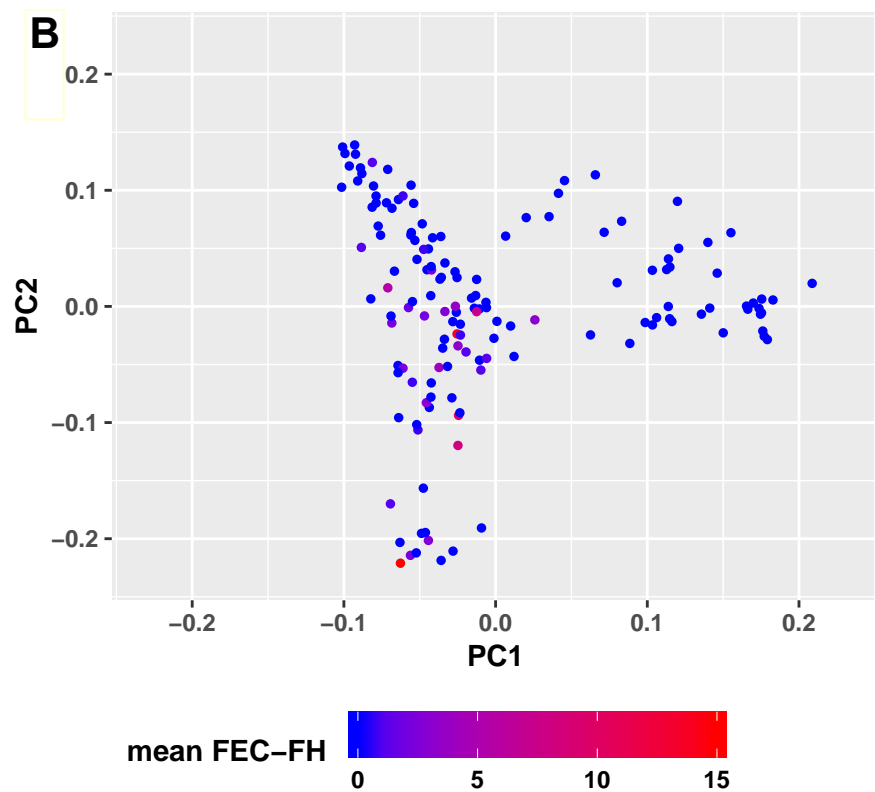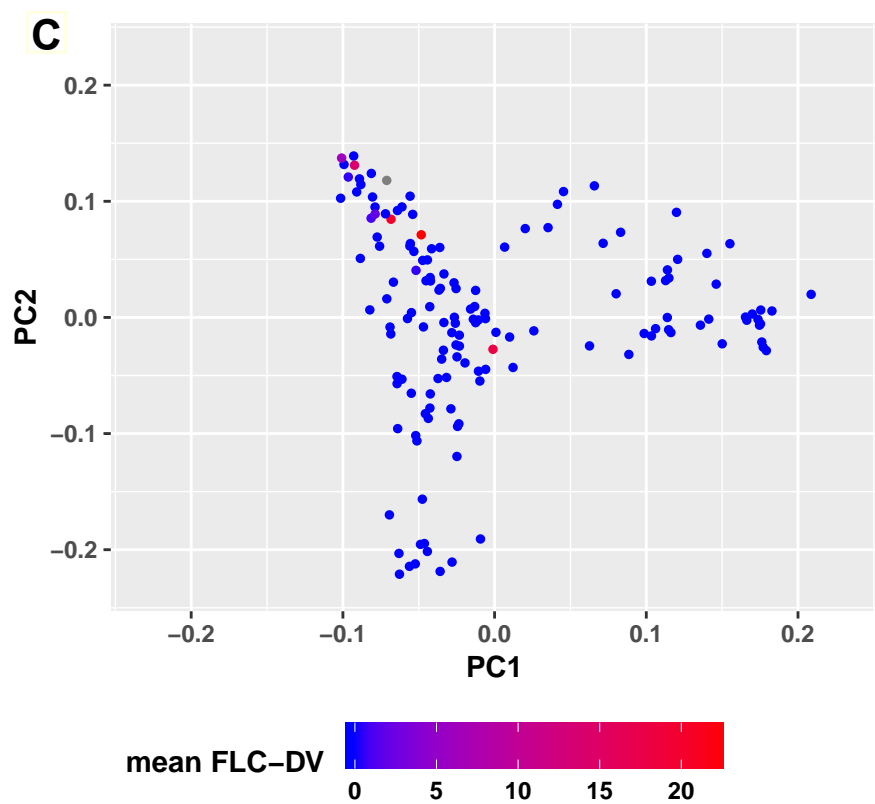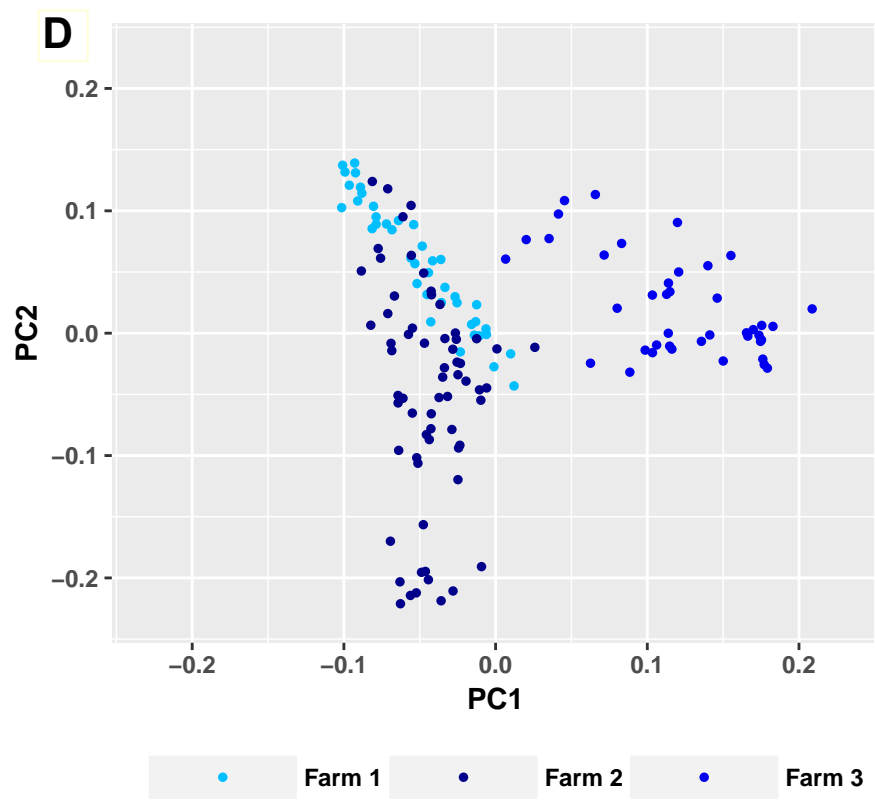

Supplement: Supplementary file 2 — Figure S1. Principal component analysis of the 148 DSN cattle for (A) the mean values of FEC-GIN, (B) the mean values of FEC-FH, (C) the mean values of FLC-DV and (D) the three different farms. Plot of the first two principal components (PC1 and PC2) of each individual cow based on SNP information to evaluate the extent of the population structure. (PDF 31 kb) [file 12864_2019_5659_MOESM2_ESM.pdf]
