# Supplementary material for: Genome-wide associations and functional gene analyses for endoparasite resistance in an endangered population of native German Black Pied cattle
Source: BMC Genomics. 2019 Apr 8;20:277. doi: 10.1186/s12864-019-5659-4 (PMC6454736; doi:10.1186/s12864-019-5659-4)
Supplement: Supplementary file 3 — Table S2. List of all SNP markers associated with the residuals of gastrointestinal nematodes (rFEC-GIN) identified in Black Pied dairy cattle by genome-wide analysis. (DOCX 19 kb) [file 12864_2019_5659_MOESM3_ESM.docx]

Additional file 3. List of all SNP markers associated with the residuals of gastrointestinal nematodes (rFEC-GIN) identified in Black Pied dairy cattle by genome-wide analysis.

| BTA | SNP name | Position (bp) | SNP effect | SE | *p*-value |
| --- | --- | --- | --- | --- | --- |
| 2 | *rs135675074* | 94,171,783 | 33.14 | 6.99 | 2.12 x 10^-6^ |
|  | *rs132921643* | 94,172,533 | 33.14 | 6.99 | 2.12 x 10^-6^ |
|  | *rs137012736* | 94,174,089 | 33.14 | 6.99 | 2.12 x 10^-6^ |
|  | *rs135373903* | 94,176,421 | 33.14 | 6.99 | 2.12 x 10^-6^ |
| 4 | *rs134978883* | 2,906,710 | 27.15 | 6.02 | 6.38 x 10^-6^ |
|  | *rs111009671* | 94,017,547 | 32,51 | 6,83 | 1.97 x 10^-6^ |
| 5 | *rs43427386* | 7,162,997 | 33.23 | 7.17 | 3.59 x 10^-6^ |
| 8 | *rs110654845* | 16,726,638 | 27.17 | 5.97 | 5.26 x 10^-6^ |
| 9 | *rs42686248* | 55,913,164 | 21.90 | 4.76 | 4.27 x 10^-6^ |
|  | *rs42684203* | 55,982,484 | 21.90 | 4.76 | 4.27 x 10^-6^ |
| 18 | *rs136760652* | 16,111,659 | 33.08 | 7.06 | 2.79 x 10^-6^ |
|  | *rs133189711* | 16,201,169 | 29.46 | 6.57 | 7.28 x 10^-6^ |
|  | *rs41866588* | 16,920,014 | 30.23 | 6.62 | 5.02 x 10^-6^ |
| 22 | *rs110780876* | 992,265 | 34.75 | 7.70 | 6.41 x 10^-6^ |
| 24 | *rs135792391* | 61,565,663 | 29.51 | 6.69 | 1.02 x 10^-5^ |
|  | *rs134104638* | 61,567,323 | 29.51 | 6.69 | 1.02 x 10^-5^ |
| 26 | *rs42088089* | 19,577,461 | 28.33 | 6.63 | 1.93 x 10^-5^ |
