# Supplementary material for: Genome-wide associations and functional gene analyses for endoparasite resistance in an endangered population of native German Black Pied cattle
Source: BMC Genomics. 2019 Apr 8;20:277. doi: 10.1186/s12864-019-5659-4 (PMC6454736; doi:10.1186/s12864-019-5659-4)
Supplement: Supplementary file 4 — Table S3. List of all SNP markers associated with the residuals of Fasciola hepatica (rFEC-FH) identified in Black Pied dairy cattle by genome-wide analysis. (DOCX 15 kb) [file 12864_2019_5659_MOESM4_ESM.docx]

Additional file 4. List of all SNP markers associated with the residuals of *Fasciola hepatica* (rFEC-FH) identified in Black and White dairy cattle by genome-wide analysis.

| BTA | SNP name | Position (bp) | SNP effect | SE | *p*-value |
| --- | --- | --- | --- | --- | --- |
| 1 | *rs110835791* | 50,365,140 | 1.56 | 0.34 | 5.38 x 10^-6^ |
| 7 | *rs41664083* | 94,639,822 | 1.77 | 0.34 | 2.59 x 10^-7^ |
|  | *rs135348628* | 94,745,593 | 1.77 | 0.34 | 2.59 x 10^-7^ |
|  | *rs136054186* | 111,644,874 | 2.28 | 0.45 | 4.15 x 10^-7^ |
|  | *rs135096708* | 111,643,709 | 2.04 | 0.43 | 2.69 x 10^-6^ |
| 28 | *rs133005080* | 38,500,927 | 2.27 | 0.53 | 1.81 x 10^-5^ |
