# Supplementary material for: Genome-wide associations and functional gene analyses for endoparasite resistance in an endangered population of native German Black Pied cattle
Source: BMC Genomics. 2019 Apr 8;20:277. doi: 10.1186/s12864-019-5659-4 (PMC6454736; doi:10.1186/s12864-019-5659-4)
Supplement: Supplementary file 5 — Table S4. List of all SNP markers associated with the residuals of Dictyocaulus viviparus (rFLC-DV) identified in Black Pied dairy cattle by genome-wide analysis. (DOCX 38 kb) [file 12864_2019_5659_MOESM5_ESM.docx]

Additional file 5. List of all SNP markers associated with the residuals of *Dictyocaulus viviparus* (rFLC-DV) identified in Black and White dair*y* cattle by genome-wide analysis.

| BTA | SNP name | Position (bp) | SNP effect | SE | *p*-value |
| --- | --- | --- | --- | --- | --- |
| 2 | *rs133101212* | 113,205,475 | 8.92 | 1.48925 | 2.10 x10^-9^ |
|  | *rs43321467* | 113,207,239 | 8.92 | 1.48925 | 2.10 x 10^-9^ |
|  | *rs43321471* | 113,210,047 | 8.92 | 1.48925 | 2.10 x 10^-9^ |
|  | *rs133768435* | 113,210,910 | 8.92 | 1.48925 | 2.10 x 10^-9^ |
|  | *rs135251342* | 113,213,058 | 8.92 | 1.48925 | 2.10 x 10^-9^ |
|  | *rs43321517* | 113,223,952 | 8.92 | 1.48925 | 2.10 x 10^-9^ |
|  | *rs43323482* | 113,291,934 | 8.92 | 1.48925 | 2.10 x 10^-9^ |
|  | *rs43323483* | 113,293,169 | 7.28 | 1.35637 | 7.95 x 10^-8^ |
|  | *rs41645202* | 112,993,253 | 7.044 | 1.33595 | 1.35 x 10^-7^ |
|  | *rs134650179* | 113,478,686 | 6.34 | 1.27459 | 6.59 x 10^-7^ |
|  | *rs110218139* | 113,024,164 | 4.00 | 0.843492 | 2.14 x 10^-6^ |
|  | *rs133748186* | 113,486,143 | 5.82 | 1.25756 | 3.66 x 10^-6^ |
| 3 | *rs43365726* | 112,096,061 | 5.90 | 1.30207 | 5.84 x 10^-6^ |
|  | *rs43366333* | 112,118,311 | 5.90 | 1.30207 | 5.84 x 10^-6^ |
|  | *rs43366338* | 112,124,938 | 5.90 | 1.30207 | 5.84 x 10^-6^ |
| 5 | *rs43422549* | 6,916,388 | 4.40 | 0.819554 | 7.69 x 10^-8^ |
|  | *rs42020386* | 1,736,633 | 4.56 | 0.900503 | 4.12 x 10^-7^ |
|  | *rs136770833* | 97,265,477 | 5.10 | 1.04053 | 9.46 x 10^-7^ |
|  | *rs133601179* | 1,562,117 | 7.03 | 1.44225 | 1.07 x 10^-6^ |
|  | *rs137416358* | 1,566,129 | 7.03 | 1.44225 | 1.07 x 10^-6^ |
|  | *rs133958808* | 1,572,027 | 7.03 | 1.44225 | 1.07 x 10^-6^ |
|  | *rs133844828* | 65,424,614 | 5.81 | 1.19985 | 1.29 x 10^-6^ |
|  | *rs110472459* | 68,420,453 | 4.60 | 0.953276 | 1.42 x 10^-6^ |
|  | *rs137016919* | 6,914,668 | 3.32 | 0.724193 | 4.69 x 10^-6^ |
|  | *rs109593883* | 68,355,562 | 5.40 | 1.18516 | 5.28 x 10^-6^ |
|  | *rs109627293* | 68,366,616 | 5.40 | 1.18516 | 5.28 x 10^-6^ |
|  | *rs134847963* | 68,374,385 | 5.40 | 1.18516 | 5.28 x 10^-6^ |
|  | *rs109729704* | 68,383,243 | 5.40 | 1.18516 | 5.28 x 10^-6^ |
|  | *rs133229007* | 97,105,569 | 3.60 | 0.797513 | 6.46 x 10^-6^ |
|  | *rs136683052* | 33,889,721 | 5.53 | 1.23422 | 7.33 x 10^-6^ |
|  | *rs135121769* | 101,571,894 | 4.50 | 1.0082 | 8.13 x 10^-6^ |
|  | *rs134744846* | 101,585,766 | 4.50 | 1.0082 | 8.13 x 10^-6^ |
|  | *rs137233679* | 101,596,166 | 4.50 | 1.01 | 8.13 x 10^-6^ |
|  | *rs136997456* | 101,618,256 | 4.50 | 1.01 | 8.13 x 10^-6^ |
| 8 | *rs136262795* | 35,573,747 | 7.69 | 1.46 | 1.48 x 10^-7^ |
|  | *rs137043944* | 35,553,300 | 7.27 | 1.42 | 3.08 x 10^-7^ |
|  | *rs41850891* | 35,564,838 | 7.27 | 1.42 | 3.08 x 10^-7^ |
|  | *rs41850898* | 35,568,857 | 7.27 | 1.42 | 3.08 x 10^-7^ |
|  | *rs136155930* | 35,494,872 | 6.81 | 1.39 | 1.00 x 10^-6^ |
|  | *rs137383802* | 35,597,508 | 6.81 | 1.39 | 1.00 x 10^-6^ |
|  | *rs134324239* | 35,604,477 | 6.81 | 1.39 | 1.00 x 10^-6^ |
|  | *rs132758433* | 35,550,656 | 6.33 | 1.33 | 1.84 x 10^-6^ |
|  | *rs110106657* | 35,565,838 | 6.33 | 1.33 | 1.84 x 10^-6^ |
| 10 | *rs134196410* | 94,003,818 | 6.39 | 1.41 | 6.20 x 10^-6^ |
| 15 | *rs41750400* | 8,975,191 | 7.63 | 1.36 | 2.06 x 10^-8^ |
|  | *rs41750406* | 8,979,052 | 7.63 | 1.36 | 2.06 x 10^-8^ |
|  | *rs41750419* | 8,984,238 | 7.63 | 1.36 | 2.06 x 10^-8^ |
|  | *rs134665586* | 8,984,833 | 7.63 | 1.36 | 2.06 x 10^-8^ |
|  | *rs41750421* | 8,985,944 | 7.63 | 1.36 | 2.06 x 10^-8^ |
|  | *rs41750428* | 8,986,630 | 7.63 | 1.36 | 2.06 x 10^-8^ |
|  | *rs41751433* | 8,987,788 | 7.63 | 1.36 | 2.06 x 10^-8^ |
|  | *rs41751439* | 8,988,625 | 7.63 | 1.36 | 2.06 x 10^-8^ |
|  | *rs41751444* | 8,990,016 | 7.63 | 1.36 | 2.06 x 10^-8^ |
|  | *rs41751452* | 8,992,789 | 7.63 | 1.36 | 2.06 x 10^-8^ |
|  | *rs109581724* | 9,025,678 | 7.63 | 1.36 | 2.06 x 10^-8^ |
|  | *rs41749574* | 9,033,366 | 7.63 | 1.36 | 2.06 x 10^-8^ |
|  | *rs41749567* | 9,035,157 | 7.63 | 1.36 | 2.06 x 10^-8^ |
|  | *rs110430504* | 9,037,212 | 7.63 | 1.36 | 2.06 x 10^-8^ |
|  | *rs110968108* | 8,925,026 | 6.65 | 1.39 | 1.70 x 10^-6^ |
|  | *rs110517446* | 8,925,594 | 6.65 | 1.39 | 1.70 x 10^-6^ |
| 17 | *rs109427713* | 19,991,734 | 7.37 | 1.36 | 6.53 x 10^-8^ |
|  | *rs29013623* | 28,168,540 | 6.35 | 1.34 | 2.21 x 10^-6^ |
| 18 | *rs41570068* | 6,621,405 | 5.16 | 1.16 | 8.20 x 10^-6^ |
| 21 | *rs41639420* | 12,918,304 | 7.64 | 1.46 | 1.73 x 10^-7^ |
|  | *rs110352059* | 12,793,997 | 6.81 | 1.38 | 8.66 x 10^-7^ |
|  | *rs109220319* | 12,908,299 | 5.13 | 1.09 | 2.59 x 10^-6^ |
|  | *rs136550449* | 70,243,037 | 6.09 | 1.30 | 2.62 x 10^-6^ |
|  | *rs134168598* | 70,244,504 | 5.82 | 1.27 | 4.49 x 10^-6^ |
|  | *rs110352830* | 32,113,699 | 6.04 | 1.36 | 8.56 x 10^-6^ |
|  | *rs134797073* | 321,19,934 | 6.04 | 1.36 | 8.56 x 10^-6^ |
|  | *rs135714468* | 32,123,869 | 6.04 | 1.36 | 8.56 x 10^-6^ |
|  | *rs136760499* | 32,127,160 | 6.04 | 1.36 | 8.56 x 10^-6^ |
|  | *rs133361420* | 32,140,037 | 6.04 | 1.36 | 8.56 x 10^-6^ |
|  | *rs133525823* | 32,160,272 | 6.04 | 1.36 | 8.56 x 10^-6^ |
|  | *rs134517799* | 32,180,070 | 6.04 | 1.36 | 8.56 x 10^-6^ |
|  | *rs137832357* | 32,184,897 | 6.04 | 1.36 | 8.56 x 10^-6^ |
|  | *rs135757504* | 32,203,110 | 6.04 | 1.36 | 8.56 x 10^-6^ |
|  | *rs133802702* | 32,212,044 | 6.04 | 1.36 | 8.56 x 10^-6^ |
|  | *rs137499414* | 32,214,988 | 6.04 | 1.36 | 8.56 x 10^-6^ |
|  | *rs134322129* | 32,243,761 | 6.04 | 1.36 | 8.56 x 10^-6^ |
|  | *rs135506397* | 32,248,881 | 6.04 | 1.36 | 8.56 x 10^-6^ |
|  | *rs134509449* | 32,257,369 | 6.04 | 1.36 | 8.56 x 10^-6^ |
|  | *rs137664985* | 32,260,181 | 6.04 | 1.36 | 8.56 x 10^-6^ |
|  | *rs137266496* | 32,266,753 | 6.04 | 1.36 | 8.56 x 10^-6^ |
|  | *rs136416616* | 32,277,971 | 6.04 | 1.36 | 8.56 x 10^-6^ |
|  | *rs133785424* | 32,285,046 | 6.04 | 1.36 | 8.56 x 10^-6^ |
|  | *rs136698863* | 32,307,624 | 6.04 | 1.36 | 8.56 x 10^-6^ |
|  | *rs137442229* | 32,317,793 | 6.04 | 1.36 | 8.56 x 10^-6^ |
|  | *rs136863366* | 32,355,723 | 6.04 | 1.36 | 8.56 x 10^-6^ |
|  | *rs133438590* | 32,383,742 | 6.04 | 1.36 | 8.56 x 10^-6^ |
|  | *rs111011829* | 32,419,140 | 6.04 | 1.36 | 8.56 x 10^-6^ |
|  | *rs134601812* | 32,462,382 | 6.04 | 1.36 | 8.56 x 10^-6^ |
|  | *rs133284553* | 32,484,660 | 6.04 | 1.36 | 8.56 x 10^-6^ |
|  | *rs132774917* | 32,551,098 | 6.04 | 1.36 | 8.56 x 10^-6^ |
|  | *rs137048883* | 32,552,338 | 6.04 | 1.36 | 8.56 x 10^-6^ |
|  | *rs135901867* | 32,553,982 | 6.04 | 1.36 | 8.56 x 10^-6^ |
|  | *rs134638377* | 32,556,030 | 6.04 | 1.36 | 8.56 x 10^-6^ |
|  | *rs41974882* | 32,580,916 | 6.04 | 1.36 | 8.56 x 10^-6^ |
|  | *rs110780898* | 32,589,381 | 6.04 | 1.36 | 8.56 x 10^-6^ |
|  | *rs41974889* | 32,602,384 | 6.04 | 1.36 | 8.56 x 10^-6^ |
| 22 | *rs110949408* | 19,159,796 | 6.55 | 1.46 | 7.31 x 10^-6^ |
| 24 | *rs132977752* | 9,694,150 | 7.96 | 1.20 | 3.77 x 10^-11^ |
|  | *rs134111269* | 8,077,784 | 5.30 | 0.89 | 2.71 x 10^-9^ |
|  | *rs133375229* | 8,089,513 | 5.30 | 0.89 | 2.71 x 10^-9^ |
|  | *rs137322379* | 8,118,143 | 5.79 | 1.00 | 7.34 x 10^-9^ |
|  | *rs110828071* | 26,974,278 | 7.06 | 1.26 | 2.07 x 10^-8^ |
|  | *rs109177598* | 22,360,773 | 5.41 | 0.97 | 2.70 x 10^-8^ |
|  | *rs41645744* | 8,071,750 | 4.64 | 0.84 | 3.36 x 10^-8^ |
|  | *rs137780857* | 16,998,232 | 5.94 | 1.09 | 4.89 x 10^-8^ |
|  | *rs136886816* | 17,021,421 | 5.94 | 1.09 | 4.89 x 10^-8^ |
|  | *rs133867370* | 6,336,382 | 6.54 | 1.26 | 1.90 x 10^-7^ |
|  | *rs134267948* | 27,709,482 | 6.64 | 1.42 | 2.89 x 10^-6^ |
|  | *rs108957431* | 21,676,840 | 4.71 | 1.04 | 5.41 x 10^-6^ |
|  | *rs111023415* | 21,681,981 | 4.71 | 1.04 | 5.41 x 10^-6^ |
|  | *rs137002683* | 29,087,857 | 3.70 | 0.83 | 9.04 x 10^-6^ |
|  | *rs137823178* | 21,629,240 | 4.28 | 0.97 | 1.09 x 10^-5^ |
|  | *rs109096413* | 9,652,945 | 6.50 | 1.48 | 1.13 x 10^-5^ |
|  | *rs110649362* | 26,510,275 | 5.71 | 1.31 | 1.26 x 10^-5^ |
|  | *rs136230042* | 8,122,526 | 5.64 | 1.30 | 1.45 x 10^-5^ |
|  | *rs109900196* | 11,953,955 | 5.36 | 1.24 | 1.53 x 10^-5^ |
|  | *rs109345412* | 6,182,494 | 5.79 | 1.34 | 1.59 x 10^-5^ |
| 26 | *rs109240510* | 4,781,510 | 5.73 | 1.27 | 6.77 x 10^-6^ |
| 29 | *rs133088719* | 13,252,956 | 6.34 | 1.48 | 1.75 x 10^-5^ |
|  | *rs136992575* | 13,254,130 | 6.34 | 1.48 | 1.75 x 10^-5^ |
|  | *rs109000169* | 132,55,568 | 6.34 | 1.48 | 1.75 x 10^-5^ |
|  | *rs109728884* | 13,257,135 | 6.34 | 1.48 | 1.75 x 10^-5^ |
|  | *rs137550236* | 13,268,747 | 6.34 | 1.48 | 1.75 x 10^-5^ |
|  | *rs110061751* | 13,273,614 | 6.34 | 1.48 | 1.75 x 10^-5^ |
|  | *rs110294632* | 13,274,592 | 6.34 | 1.48 | 1.75 x 10^-5^ |
|  | *rs136134712* | 13,276,754 | 6.34 | 1.48 | 1.75 x 10^-5^ |
|  | *rs110849642* | 13,277,661 | 6.34 | 1.48 | 1.75 x 10^-5^ |
|  | *rs109056182* | 13,279,443 | 6.34 | 1.48 | 1.75 x 10^-5^ |
|  | *rs133909880* | 13,285,311 | 6.34 | 1.48 | 1.75 x 10^-5^ |
|  | *rs137446998* | 13,286,538 | 6.34 | 1.48 | 1.75 x 10^-5^ |
|  | *rs109702906* | 13,288,322 | 6.34 | 1.48 | 1.75 x 10^-5^ |
|  | *rs110348590* | 13,293,156 | 6.34 | 1.48 | 1.75 x 10^-5^ |
|  | *rs110428552* | 13,307,214 | 6.34 | 1.48 | 1.75 x 10^-5^ |
|  | *rs109978220* | 13,311,063 | 6.34 | 1.48 | 1.75 x 10^-5^ |
|  | *rs109888653* | 13,329,371 | 6.34 | 1.48 | 1.75 x 10^-5^ |
|  | *rs109737439* | 13,330,150 | 6.34 | 1.48 | 1.75 x 10^-5^ |
|  | *rs109396565* | 13,332,265 | 6.34 | 1.48 | 1.75 x 10^-5^ |
|  | *rs108986441* | 13,333,756 | 6.34 | 1.48 | 1.75 x 10^-5^ |
|  | *rs110498051* | 13,335,055 | 6.34 | 1.48 | 1.75 x 10^-5^ |
|  | *rs134834406* | 13,335,947 | 6.34 | 1.48 | 1.75 x 10^-5^ |
|  | *rs110708167* | 13,339,550 | 6.34 | 1.48 | 1.75 x 10^-5^ |
|  | *rs109690005* | 13,342,301 | 6.34 | 1.48 | 1.75 x 10^-5^ |
|  | *rs109863863* | 13,343,481 | 6.34 | 1.48 | 1.75 x 10^-5^ |
|  | *rs110168180* | 13,346,362 | 6.34 | 1.48 | 1.75 x 10^-5^ |
|  | *rs132779127* | 13,351,796 | 6.34 | 1.48 | 1.75 x 10^-5^ |
|  | *rs110080951* | 13,361,612 | 6.34 | 1.48 | 1.75 x 10^-5^ |
|  | *rs109562572* | 13,370,202 | 6.34 | 1.48 | 1.75 x 10^-5^ |
|  | *rs110243030* | 13,371,914 | 6.34 | 1.48 | 1.75 x 10^-5^ |
|  | *rs110760342* | 13,379,969 | 6.34 | 1.48 | 1.75 x 10^-5^ |
|  | *rs135432702* | 13,388,874 | 6.34 | 1.48 | 1.75 x 10^-5^ |
|  | *rs41621467* | 13,392,044 | 6.34 | 1.48 | 1.75 x 10^-5^ |
|  | *rs109167255* | 13,393,654 | 6.34 | 1.48 | 1.75 x 10^-5^ |
|  | *rs110816500* | 13,401,385 | 6.34 | 1.48 | 1.75 x 10^-5^ |
|  | *rs136184785* | 13,414,522 | 6.34 | 1.48 | 1.75 x 10^-5^ |
|  | *rs109526288* | 13,439,573 | 6.34 | 1.48 | 1.75 x 10^-5^ |
|  | *rs133454249* | 13,443,333 | 6.34 | 1.48 | 1.75 x 10^-5^ |
|  | *rs109611361* | 13,456,917 | 6.34 | 1.48 | 1.75 x 10^-5^ |
|  | *rs42163709* | 13,509,419 | 6.34 | 1.48 | 1.75 x 10^-5^ |
|  | *rs109048330* | 13,553,384 | 6.34 | 1.48 | 1.75 x 10^-5^ |
|  | *rs133875991* | 13,559,340 | 6.34 | 1.48 | 1.75 x 10^-5^ |
|  | *rs110349107* | 13,560,884 | 6.34 | 1.48 | 1.75 x 10^-5^ |
|  | *rs132633317* | 13,562,508 | 6.34 | 1.48 | 1.75 x 10^-5^ |
